# Brain Stem Death as the Vital Determinant for Resumption of Spontaneous Circulation after Cardiac Arrest in Rats

**DOI:** 10.1371/journal.pone.0007744

**Published:** 2009-11-04

**Authors:** Alice Y. W. Chang, Julie Y. H. Chan, Yao-Chung Chuang, Samuel H. H. Chan

**Affiliations:** 1 Center for Translational Research in Biomedical Sciences, Chang Gung Memorial Hospital-Kaohsiung Medical Center, Kaohsiung County, Taiwan, Republic of China; 2 Center for Neuroscience, National Sun Yat-sen University, Kaohsiung, Taiwan, Republic of China; 3 Department of Medical Education and Research, Kaohsiung Veterans General Hospital, Kaohsiung, Taiwan, Republic of China; 4 Department of Neurology, Chang Gung Memorial Hospital-Kaohsiung Medical Center, Chang Gung University College of Medicine, Kaohsiung County, Taiwan, Republic of China; Julius-Maximilians-Universität Würzburg, Germany

## Abstract

**Background:**

Spontaneous circulation returns to less than half of adult cardiac arrest victims who received in-hospital resuscitation. One clue for this disheartening outcome arises from the prognosis that asystole invariably takes place, after a time lag, on diagnosis of brain stem death. The designation of brain stem death as the point of no return further suggests that permanent impairment of the brain stem cardiovascular regulatory machinery precedes death. It follows that a crucial determinant for successful revival of an arrested heart is that spontaneous circulation must resume before brain stem death commences. Here, we evaluated the hypothesis that maintained functional integrity of the rostral ventrolateral medulla (RVLM), a neural substrate that is intimately related to brain stem death and central circulatory regulation, holds the key to the vital time-window between cardiac arrest and resumption of spontaneous circulation.

**Methodology/Principal Findings:**

An animal model of brain stem death employing the pesticide mevinphos as the experimental insult in Sprague-Dawley rats was used. Intravenous administration of lethal doses of mevinphos elicited an abrupt cardiac arrest, accompanied by elevated systemic arterial pressure and anoxia, augmented neuronal excitability and enhanced microvascular perfusion in RVLM. This period represents the vital time-window between cardiac arrest and resumption of spontaneous circulation in our experimental model. Animals with restored spontaneous circulation exhibited maintained neuronal functionality in RVLM beyond this critical time-window, alongside resumption of baseline tissue oxygen and enhancement of local blood flow. Intriguingly, animals that subsequently died manifested sustained anoxia, diminished local blood flow, depressed mitochondrial electron transport activities and reduced ATP production, leading to necrotic cell death in RVLM. That amelioration of mitochondrial dysfunction and bioenergetic failure in RVLM by coenzyme Q10, the mobile electron carrier in mitochondrial respiratory chain, or oxygenation restored spontaneous circulation further established a causal relationship between functionality of RVLM and resumed spontaneous circulation after cardiac arrest.

**Conclusions/Significance:**

We conclude that whereas necrotic cell death because of bioenergetic failure triggered by anoxia in RVLM, which precipitates brain stem death, negates resuscitation of an arrested heart, maintained functional integrity of this neural substrate holds the key to resumption of spontaneous circulation after cardiac arrest in rats.

## Introduction

Two most daunting and unresolved medical enigmas in contemporary medicine are resumption of spontaneous circulation after cardiac arrest and brain stem death. This article argues that those two seemingly disparate clinical events are in fact intertwined, and together offer a novel explanation for the disheartening observation that spontaneous circulation returns to only 44% of 14,720 adult cardiac arrest victims who received in-hospital resuscitation [1].

The underlying premise for the time-honored practice of cardiac resuscitation is that an arrested heart may still be revived. In the context of brain stem death as the legal definition of death [2]–[5], however, a person is dead even with a beating heart. Our first clue that these two eventualities are interrelated arises from the prognosis that asystole invariably takes place, after a time lag, on diagnosis of brain stem death [5]. The designation of brain stem death as the point of no return [6] further suggests that permanent impairment of the brain stem cardiovascular regulatory machinery precedes the inevitable asystole [7]. The identification by our laboratory of a common denominator among critically ill patients who died offers another crucial clue. In comatose patients who succumbed to systemic inflammatory response syndrome [8], severe brain injury [9] or organophosphate poisoning [10], we found that the sequence of cardiovascular events before death invariably entails a dramatic reduction or loss of the low-frequency component (LF; 0.004 to 0.15 Hz) in the power spectrum of their systemic arterial pressure (SAP) signals, which reflects brain stem death [7], followed progressively by hypotension and eventually asystole. Most intriguingly, we confirmed that this life-and-death signal reflects the functional integrity of the brain stem, and is vital to the resumption of heart functions in patients rendered cardioplegic during coronary artery bypass grafting procedures [11]. An immediate corollary that arises from these clinical observations is that a crucial determinant for successful revival of an arrested heart is that spontaneous circulation must resume before brain stem death commences.

An appropriate animal model to scrutinize this notion must satisfy three basic requirements. First, the experimental insult must elicit simultaneous actions on the brain stem and the heart. Second, it should allow for concurrent determination of the time-course of the resultant cardiac arrest, manifestation of brain stem death and resumption of spontaneous circulation. Finally, it should also allow for delineation of the causal cellular mechanisms that underpin the temporal relationship thus identified between the above events. In this regard, a likely neural substrate that holds the key to the vital time-window between cardiac arrest and resumption of spontaneous circulation is the rostral ventrolateral medulla (RVLM). The RVLM is long known to play a crucial role in brain stem cardiovascular regulation [12], including maintenance of sympathetic vasomotor tone and stable SAP. Our laboratory [13] further demonstrated that this brain stem site is the origin of the life-and-death signal that invariably diminishes or disappears to reflect failure of the central cardiovascular regulatory machinery before brain stem death in critically ill patients [7]. In conjunction with this neural substrate, an appropriate model is the mevinphos (Mev) intoxication model of brain stem death [7], [14], [15] that employs this US Environmental Protection Agency Toxicity Category I pesticide as the experimental insult. The first and foremost reason is that Mev acts on the RVLM to elicit phasic cardiovascular responses [16] that reflect the waxing and waning of the life-and-death signal, the disappearance of which signifies imminent brain stem death in patients suffering from severe organophosphate poisoning [7], [10]. Most intriguingly, the disappearance of this life-and-death signal invariably takes place before significant hypotension and bradycardia. As an anticholinesterase, Mev also elicits cardiac depression by a vagomimetic action on the heart via accumulation of acetylcholine. In addition, Mev changes tissue oxygen level and blood flow in the RVLM [17]. On intravenous administration of Mev, this animal model therefore allowed us to determine the temporal relationship between the elicited cardiac arrest, alterations in SAP, the life-and-death signal and tissue oxygen level, blood flow or temperature in the RVLM. It also allowed for biochemical analyses of the cellular events that took place in the RVLM during the vital time-window that interposes between cardiac arrest and resumption of spontaneous circulation.

Based on the Mev intoxication model of brain stem death, the present study evaluated the hypothesis that the functional integrity of RVLM holds the key to the vital time-window between cardiac arrest and resumption of spontaneous circulation. In addition to validating this hypothesis, our results revealed that the repertoire of cellular events that underlies the loss of functional integrity in the RVLM, which underpins the failure of resumption of spontaneous circulation after cardiac arrest because of the precipitated brain stem death, entails progression towards sustained anoxia, cessation of local blood flow, dysfunction of mitochondrial electron transport chain and reduction in ATP production, leading to necrotic cell death.

## Materials and Methods

### Ethics Statement

All experimental procedures have been approved by the Committee on Laboratory Animals, National Sun Yat-sen University, and were carried out in accordance with their guidelines. Efforts were made to reduce the number of animals used and to minimize animal suffering during the experiment.

### Animals

Adult male Sprague-Dawley rats (276 to 322 g, n = 160) purchased from the Experimental Animal Center of the National Science Council, Taiwan, Republic of China were used. They were housed in an animal room under temperature control (24–25°C) and 12-h light-dark cycle. Standard laboratory rat chow and tap water were available ad libitum.

### General Preparation

Rats received preparatory surgery under an induction dose of intraperitoneally administered pentobarbital sodium (50 mg/kg) that included tracheal intubation and cannulation of the formal artery and vein. Animals received thereafter an intravenous infusion of propofol (Zeneca, Macclesfield, UK) at 20–25 mg/kg/h, which provided satisfactory maintenance of anesthesia while preserving the capacity of central cardiovascular regulation [18]. SAP signals recorded from the femoral artery were subject simultaneously to on-line and real-time power spectral analysis, using a computer algorithm developed by our laboratory [19] that is specifically designed to deal with non-stationary signals encountered in clinical [8]–[11] and laboratory [14]–[18] settings. We were particularly interested in the low-frequency (LF; 0.25–0.8 Hz) component in the SAP spectrum because its power density mirrors the prevalence of baroreflex-mediated sympathetic neurogenic vasomotor discharges that emanate from this brain stem site [20]. With particular reference to the present study, a significant reduction in LF power denotes loss of functional integrity of RVLM that precedes brain stem death [7]–[10]. Heart rate (HR) was derived instantaneously from SAP signals. During the experiment, animals were allowed to breathe spontaneously with room air, and body temperature was maintained at 37°C by a heating pad.

### Study Design

We used the Mev intoxication model of brain stem death [7], [14], [15] to determine the temporal alterations elicited by an intravenous administration of Mev in SAP or HR, and correlated them with the concurrent changes in neuronal functionality, tissue oxygen level, blood flow or temperature in the RVLM. We also carried out biochemical analyses of the cellular events (see below) that took place in the RVLM during the vital time-window that interposes between cardiac arrest and resumption of spontaneous circulation.

### Measurement of Tissue Oxygen Level, Microvascular Perfusion and Temperature

As in our previous study [17], a combined oxygen/temperature/blood flow probe designed for simultaneous and continuous measurement of tissue oxygen tension, blood flow and temperature (Oxford Optronix, Oxford, England) was stereotaxically positioned into the RVLM. The coordinates used were: 4.5 to 5 mm posterior to the lambda, 1.8 to 2.1 mm lateral to the midline and 8.1 to 8.4 mm below the dorsal surface of the cerebellum. The tip of the probe has a dimension of approximately 800 µm. Instantaneous changes in local oxygen tension, compensated for fluctuations in tissue temperature, were processed by an OxyLite monitor (Oxford Optronix). Real-time microvascular red blood cell perfusion in tissue was processed by an OxyFlo monitor (Oxford Optronix). The laser Doppler signals from the tissue were recorded in blood perfusion units (BPU), which is a relative unit defined against a controlled motility standard.

### Assay for Mitochondrial Respiratory Enzyme Activities

Tissues on both sides of the ventrolateral part of medulla oblongata, at the level of the RVLM (0.5–2.5 mm rostral to the obex), were collected by micropunches made with a 1 mm (i.d.) stainless steel bore [14] immediately after animals died of Mev intoxication or as soon as spontaneous circulation returned to baseline after Mev-induced cardiac arrest. Samples of ventrolateral medulla collected from rats that were anesthetized but without receiving further experimental manipulations served as the sham control. Isolation of mitochondria and analysis of mitochondrial electron transport enzyme activities were performed as reported previously [17]. As a routine, we determined the activity of nicotinamide adenine dinucleotide (NADH) cytochrome c reductase (NCCR; marker enzyme for electron transport capacity between Complexes I and III), succinate cytochrome c reductase (SCCR; marker enzyme for electron transport capacity between Complexes II and III) or cytochrome c oxidase (CCO; marker enzyme for Complex IV). At least quadruplicate determination was carried out for each tissue sample in all enzyme assays. Total protein in the mitochondrial suspension was estimated using a protein assay kit (Pierce, Rockford, IL).

### Measurement of ATP Concentration

Samples of the ventrolateral medulla were processed for determination of ATP concentration as described previously [17], using an ATP bioluminescence assay (Roche Diagnostics GmbH, Mannheim, Germany). Light emitted from a luciferase-mediated reaction and measured by a tube luminometer (Berthold Detection Systems GmbH, Pforzheim, Germany) was used to calculate the measured values.

### Electron Microscopy

Brain stem tissues that contained the RVLM collected immediately after animals died of Mev-induced cardiac arrest or from sham-controls were diced and submerged in 4% glutaraldehyde (0.1 M sodium cacodylate buffer, pH 7.2). Tissues were post-fixed with osmium, and en bloc stained with uranyl acetate. After dehydration, each specimen was embedded by infiltration in Spurr's medium. Following trimming of the tissue blocks, sections were cut to a thickness of 90 nm, post-stained with uranyl acetate and lead citrate, and viewed on 300 mesh-coated grids using a JEOL JEM-2000 EXII (Tokyo, Japan) electron microscope. Approximately 100 micrographs from each specimen were examined by a pathologist in a blind manner to eliminate bias in data interpretation.

### Pretreatments

Pretreatments with a highly mobile electron carrier between the flavoproteins and cytochromes in the mitochondrial respiratory chain [21] and a potent antioxidant [22], coenzyme Q10 (CoQ10; kindly provided by Dr. Marianna Sikorska of National Research Council, Ottawa, Canada) was either administered intravenously or microinjected bilaterally into the RVLM using a stereotaxically positioned 27-gauge needle that was connected to a 0.5-µl Hamilton microsyringe (Reno, NV) [14]–[17]. The coordinates for the RVLM were the same as those used for measurement of tissue oxygen, temperature and blood flow. The volume of injection was restricted to 50 nl, and was delivered to each side of the RVLM over 1−2 min to allow for complete diffusion. Possible volume effect was controlled by injecting the same amount (50 nl) of artificial cerebrospinal fluid (aCSF). Pretreatment with oxygenation was delivered by allowing the animals to breathe 100% oxygen for 30 min.

### Statistical Analysis

All values are expressed as mean±SEM. The values of MSAP, HR, power density of LF component of SAP signals and tissue oxygen level, microvascular perfusion or temperature in the RVLM, at 1-min intervals before and after Mev administration, were tabulated. The temporal effects of various treatments on these parameters were assessed using two-way analysis of variance (ANOVA) with repeated measures for group difference. The activity of mitochondrial respiratory enzymes or ATP level in ventrolateral medulla was assessed with one-way ANOVA. In both cases, Scheffé multiple-range test was used for posteriori comparison of individual means. *P*<0.05 was considered to be statistically significant.

## Results

### Loss of Functional Integrity of RVLM Prevents Resumption of Spontaneous Circulation after Cardiac Arrest

**Figure 1:**
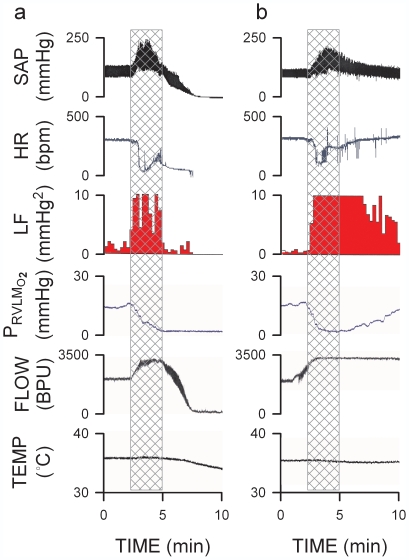
Loss of functional integrity of rostral ventrolateral medulla (RVLM) because of anoxia prevents resumption of spontaneous circulation after cardiac arrest. Representative continuous tracings showing temporal changes in systemic arterial pressure (SAP), heart rate (HR), power density of low-frequency (LF) component of SAP spectrum as an experimental index for functional integrity of RVLM and prevalence of the “life-and-death” signal, and tissue oxygen concentration, microvascular perfusion or temperature in RVLM of rats that died of (a) or resumed spontaneous circulation after (b) cardiac arrest induced by intravenous administration of mevinphos (Mev; 1000 µg/kg; at time zero). The vital time-window between cardiac arrest and resumption of spontaneous circulation during which anoxia took place in RVLM is demarcated by crossed bars.

**Figure 2:**
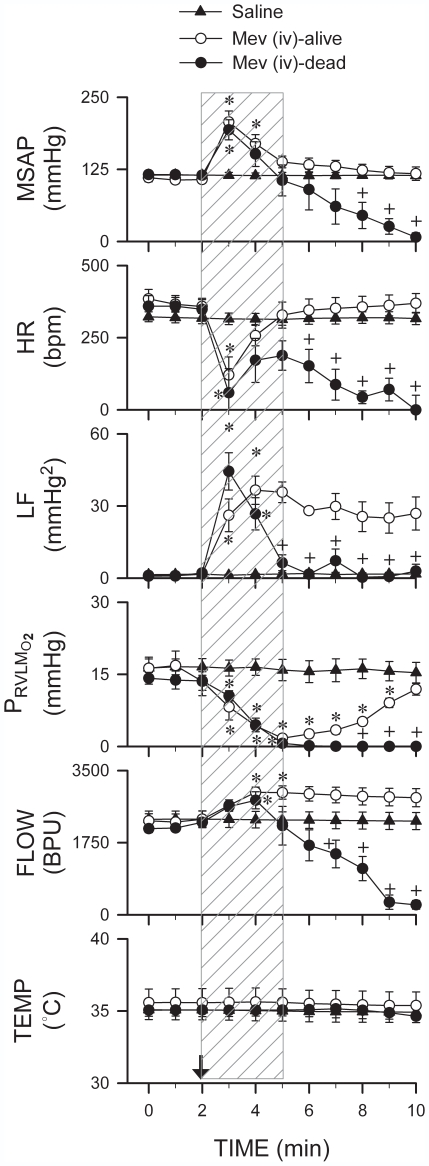
Loss of functional integrity because of anoxia in RVLM prevents resumption of spontaneous circulation after cardiac arrest. Temporal changes in mean SAP (MSAP), HR, power density of LF component of SAP spectrum, and tissue oxygen concentration, microvascular perfusion or temperature in RVLM of rats that survived or died of cardiac arrest induced by intravenous administration of Mev (1000 µg/kg; at arrow). Again, the vital time-window between cardiac arrest and resumption of spontaneous circulation during which anoxia took place in RVLM is demarcated by hatched bars. Values are mean±SEM of 5–7 animals per experimental group. ^*^ *p*<0.05 versus saline group, and ^+^ *p*<0.05 versus alive group at corresponding time-points in the post hoc Scheffé multiple-range analysis following two-way ANOVA.

Figures 1 and 2 present two scenarios elicited by intravenous administration of a high dose (1000 µg/kg) of Mev. Despite an abrupt and drastic bradycardia in both instances, SAP was elevated, together with a reversal in HR. There was a concomitant increase in the power density of the LF component of SAP signals that reflects heightened baroreflex-mediated sympathetic neurogenic vasomotor tone and prevalence of the “life-and-death” signal [7]. We found that this period, which typically endured 2.5 min in our animal model, represents the vital time-window between cardiac arrest and resumption of spontaneous circulation. Specifically, our results showed that whether animals subsequently manifested asystole, loss of SAP and died (Figs. 1a and 2) or exhibited resumption of spontaneous circulation (Figs. 1b and 2) depended on whether the augmented LF power persisted beyond this critical time-window.

**Figure 3:**
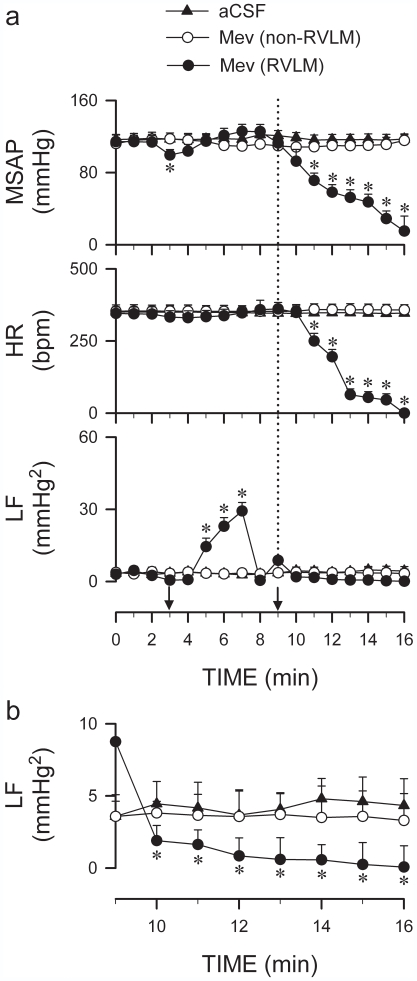
Loss of functional integrity of RVLM precedes cardiovascular depression. (a) Temporal changes in MSAP, HR or power density of LF component of SAP spectrum in rats that received consecutive microinjections (at arrows) of a lethal dose of Mev (280 nmol) bilaterally into the RVLM or sites immediately adjacent to the RVLM (non-RVLM). (b) Re-drawn of (a) between the 9^th^ [dotted line in (a)] and 16^th^ min on an expanded scale showing temporal changes in LF power. Values are mean±SEM of 5–7 animals per experimental group. ^*^ *p*<0.05 versus artificial cerebrospinal fluid (aCSF) group or non-RVLM group at corresponding time-points in the post hoc Scheffé multiple-range analysis following two-way ANOVA.

For the argument that sustained neuronal functionality in the RVLM is the crucial determinant for the resumption of spontaneous circulation after cardiac arrest to be valid, it is imperative that a loss of the life-and-death signal must precede cardiovascular depression. Figure 3 shows that this is indeed the case, taking advantage of our previous observation that Mev acts directly on the RVLM to elicit its cardiovascular effects [16]. Similar to intravenous administration (Fig. 2), microinjection unilaterally into the RVLM of a lethal dose of Mev (280 nmol) induced a transient and significant increase in the power density of the LF component that typically lasted 5 min. This life-and-death signal underwent significant reduction on subsequent application of the same dose of Mev into the contralateral RVLM. It is intriguingly to note that near disappearance of neuronal functionality in the RVLM already occurred 1 min before significant hypotension and bradycardia took place, at least 5 min before the appearance of near zero SAP and asystole. The specificity of this temporal relationship between LF power, loss of SAP, asystole and death was confirmed when microinjection bilaterally of the same dose of Mev (280 nmol) into sites adjacent to the RVLM failed to elicit these events (Fig. 3).

### Tissue Oxygen Level as a Determinant of Functional Integrity of RVLM

We found that a causal factor for the loss of neuronal functionality in the RVLM after cardiac arrest is anoxia. Animals that died (Figs. 1a and 2) exhibited progressive hypoxia in the RVLM that reached anoxic level towards the end of the crucial time-window, mirrored by an increase in local blood flow. Sustained anoxia beyond this point was accompanied by a progressive decline in microvascular perfusion to the RVLM and reduction in LF power that paralleled temporally the cessation of SAP or HR, followed by a drop in RVLM tissue temperature. On the other hand, despite an initial anoxia during the critical time-window, animals with restored SAP and HR (Figs. 1b and 2) showed sustained augmentation of power density of LF component of SAP signals, alongside a return to normal tissue oxygen level, persistent enhancement of local blood flow and maintained temperature in the RVLM.

### Bioenergetic Failure That Leads to Necrotic Cell Death Accounts for the Loss of Functional Integrity in RVLM

**Figure 4:**
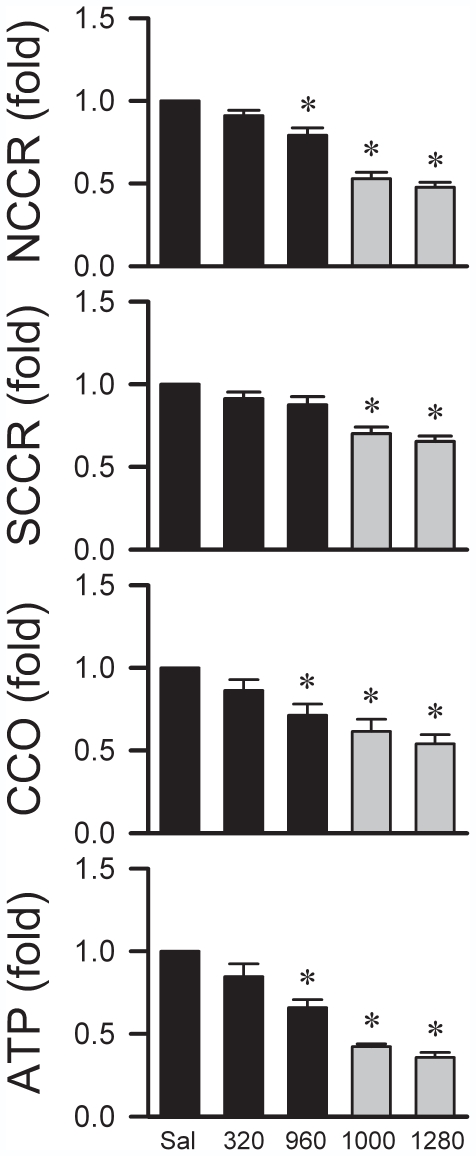
Bioenergetic failure accounts for the loss of functional integrity in RVLM. Fold changes against saline-control group in enzyme activity of NADH cytochrome c reductase (NCCR), succinate cytochrome c reductase (SCCR) or cytochrome c oxidase (CCO) and ATP production in mitochondria isolated from ventrolateral medulla of rats that received intravenous administration of Mev at sub-lethal (320 or 960 µg/kg) or lethal (1000 or 1280 µg/kg) doses. Values are mean±SEM of quadruplicate analyses from samples pooled from 5–7 animals per experimental group. ^*^ *p*<0.05 versus saline group in the post hoc Scheffé multiple-range analysis following one-way ANOVA.

Since the primary source of metabolic energy in neurons is respiratory ATP generation, neurons tend to undergo necrosis in response to anoxic stress [23]. Further results revealed that necrotic cell death as a consequence of anoxia indeed accounts for the loss of functional integrity in the RVLM beyond the critical time-window. Sub-lethal doses of Mev (320 or 960 µg/kg) that did not elicit cardiac arrest reduced the activity of NCCR, which couples mitochondrial electron transport between Complexes I and II, and CCO (Fig. 4). Lethal doses of Mev (1000 or 1280 µg/kg) that induced cardiac arrest additionally depressed the electron coupling capacity between Complexes II and III by inhibiting SCCR, leading to drastic reduction in ATP production (Fig. 4).

**Figure 5:**
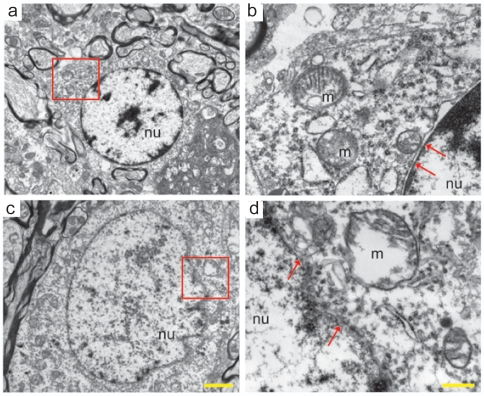
Necrotic cell death accounts for the loss of functional integrity in RVLM. Representative electron photomicrographs showing ultrastructure of RVLM neurons from sham-control animals (a and b) or animals that received intravenous administration of a lethal dose of Mev (1280 µg/kg) (c and d). Panels c and e represent enlargement of areas denoted in a and c. Note intact (b) and disrupted (d) nuclear membrane integrity (arrows); normal (a) and enlarged nucleus (nu) with nuclear electron-lucency (c); and intact (b) and swollen mitochondria (m) with disarrayed cristae (d). These results are typical of 4 animals from each experimental group. Scale bars: 2 µm in a and c; 0.5 µm in b and d.

Electron microscopy confirmed the occurrence of necrosis in the RVLM in animals that died of fatal Mev intoxication. RVLM neurons from sham-control animals showed oval nuclear morphology and normal cytoplasmic density (Fig. 5a), alongside normal mitochondrial morphology and intact nuclear membrane (Fig. 5b). On the other hand, RVLM neurons in animals that did not resume spontaneous circulation after Mev-induced cardiac arrest showed necrotic features that included enlarged nucleus, nuclear or cytoplasmic electron lucency (Fig. 5c), disrupted integrity of nuclear membrane and swollen mitochondria with disarrayed cristae (Fig. 5d).

### Bioenergetic Failure in RVLM Also Accounts for the Failure to Resume Spontaneous Circulation after Cardiac Arrest

**Figure 6:**
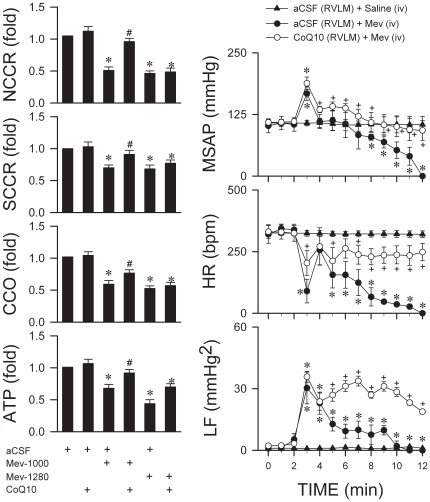
Bioenergetic failure in RVLM also accounts for the failure to resume spontaneous circulation after cardiac arrest. (Left column) Fold changes against aCSF-control group in enzyme activity of NCCR, SCCR or CCO and ATP production in mitochondria isolated from ventrolateral medulla of rats that were pretreated with microinjection bilaterally of coenzyme Q10 (CoQ10; 6 µg) or aCSF into the RVLM, followed by intravenous administration of Mev at 1000 or 1280 µg/kg. Values are mean±SEM of quadruplicate analyses from samples pooled from 5–7 animals per experimental group. ^*^ *p*<0.05 versus aCSF group and ^#^ *p*<0.05 versus corresponding aCSF+Mev group in the post hoc Scheffé multiple-range analysis following one-way ANOVA. (Right column) Also shown are effects of the CoQ10 or aCSF pretreatment on temporal changes in MSAP, HR or power density of LF component of SAP spectrum induced by intravenous (iv) administration of Mev (1000 µg/kg; at time zero) or saline. ^*^ *p*<0.05 versus aCSF+saline group and ^+^ *p*<0.05 versus aCSF+Mev group at corresponding time-points in the post hoc Scheffé multiple-range analysis following two-way ANOVA.

We further employed CoQ10, a highly mobile electron carrier between the flavoproteins and cytochromes in the mitochondrial respiratory chain [21] and a potent antioxidant [22], to establish a causal relationship between mitochondrial electron transport dysfunction, bioenergetic failure and lack of resumed spontaneous circulation after cardiac arrest. Microinjection bilaterally into RVLM of CoQ10 (6 µg) significantly reversed the depressed NCCR, SCCR or CCO activity and reduced ATP production induced by 1000 µg/kg, but not 1280 µg/kg of Mev (Fig. 6). Intriguingly, improvement of mitochondrial electron transport capacity between Complexes I and III or II and III in RVLM by CoQ10 (6 µg), similar to oxygenation, also completely resumed spontaneous circulation after cardiac arrest induced by Mev at 1000 µg/kg (Table 1). Indeed, these animals manifested a temporal pattern (Fig. 6) of sustained augmentation of LF power beyond the critical time-window, alongside maintained SAP and HR that resembled those illustrated in Figs. 1b and 2. Local application of CoQ10 to the RVLM, similar to intravenous administration of the same amount of CoQ10 (6 µg), was only partially successful against 1280 µg/kg of Mev, although oxygenation was effective (Table 1).

**Table 1:** Probability of resumption of spontaneous circulation after cardiac arrest induced by intravenous administration of Mev.

	Mev (1000 µg/kg)	Mev (1280 µg/kg)
aCSF (RVLM)	20%	0%
CoQ10 (RVLM)	100%	28%
Oxygenation	100%	100%
Saline (i.v.)	0%	0%
CoQ10 (i.v.)	33%	0%

Animals were pretreated with microinjection bilaterally of CoQ10 (6 µg) or aCSF into the RVLM, intravenous injection of CoQ10 (6 µg) or saline, or breathing 100% oxygen for 30 min. Each experimental group is made up of 5–7 animals.

## Discussion

The present study revealed that commencement of brain stem death before the resumption of spontaneous circulation after cardiac arrest offers a hitherto unavailable answer to the fateful question of why is resuscitation of an arrested heart successful in some victims but not the others. We showed that whereas maintained functional integrity of the RVLM interfaces the vital window between resumption of spontaneous circulation and cardiac arrest, necrotic cell death as a result of bioenergetic failure triggered by anoxia in the RVLM underlies brain stem death and negates cardiac resuscitation.

In a series of studies carried out in the intensive care unit, our laboratory found in critically ill patients who succumbed to either organophosphate poisoning [10], systemic inflammatory response syndrome [8] or severe brain injury [9] that a progressive and dramatic reduction or loss in the power density of the LF component of SAP signals invariably precedes death. The identification of RVLM as the origin of this spectral component [13] implies that maintained neuronal functionality in this brain stem site, and hence life-and-death, is reflected in its power density. Based on this experimental index, we were able to reveal the novel finding that a crucial determinant for successful revival of cardiac arrest is that spontaneous circulation must resume before brain stem death commences, and that maintained functional integrity of the RVLM, leading to sustained sympathetic vasomotor tone, holds the key to this vital time-window.

We found that tissue oxygen level and microvascular perfusion play a major role in the maintenance of functional integrity of the RVLM. Neuronal functions are critically dependent on a continuous supply of oxygen because their primary source of metabolic energy is oxidative phosphorylation in the mitochondrion. The mitochondrion is also the primary site of oxygen consumption in the cell, and as such presents itself as a crucial cellular contributor to brain dead because of its susceptibility to anoxia. Our results demonstrated that sub-lethal doses of Mev elicited bioenergetic failure at the RVLM by depressing only the activity of NCCR and CCO in the mitochondrial electron transport chain; SCCR activity was additionally reduced on administration of lethal doses. The implied notion that SCCR activity is a crucial determinant in the RVLM for fatality is in line with the known energy source of neurons. Aerobic metabolism of glucose is coupled to oxidative phosphorylation by linking to SCCR, FAD and Complex II through succinate in the tricarboxylic acid cycle [24]. It is conceivable that depression by anoxia of biochemical pathways that are linked to NADH and FAD, which result in irreversible reduction in intracellular ATP contents, precipitates the eventual cessation of neuronal functions in the RVLM.

A critical determinant of the eventual cell death fate resides in intracellular ATP concentration. Whereas ATP depletion is associated with necrosis, ATP is required for the development of apoptosis [23], [25], [26]. The significant depletion of intracellular ATP content in the RVLM induced by anoxia may therefore entail damage of cell membrane integrity, leading to necrotic cell death. Our results from electron microscopy support this eventuality. Since the primary source of metabolic energy in neurons is respiratory ATP generation, neurons tend to undergo necrosis in response to stress [23]. The implied notion that SCCR activity is a crucial determinant for fatality is therefore also in line with the stipulation that the ratio between glycolytic and respiratory ATP generation is proportional to apoptosis/necrosis susceptibility [23]. Of note is that hemorrhagic necrosis is detected in the brain stem of patients who died of acute organophosphate poisoning on postmortem neuropathological evaluations [27].

It is intriguing that CoQ10 assumes a protective role against lethal Mev intoxication, leading to the resumption of spontaneous circulation after cardiac arrest. We reported previously [17] that the reversal by CoQ10 of Mev-induced depression in NCCR, SCCR or CCO activity or reduction in ATP level in ventrolateral medulla is accompanied by amelioration of the sustained tissue hypoxia. The concentration of CoQ10 in the mitochondrial membrane is also related to the rate of electron transfer and respiratory function [28]. Furthermore, CoQ10 is an essential component of the mitochondrial electron transport chain [21]. It is therefore conceivable that, by enhancing quantitatively the availability of freely mobile electron carriers and qualitatively the efficacy of electron transfer across the mitochondrial respiratory chain, CoQ10 may augment the amount of tissue oxygen by reducing the leakage of electrons that form superoxide anion with oxygen. As an antioxidant [22], CoQ10 may also avail more oxygen to the neurons for oxidative phosphorylation. The resultant maintenance of functional integrity of the RVLM by these cellular actions in turn leads to the resumption of spontaneous circulation after cardiac arrest. This notion is further substantiated by results from pretreatment with oxygenation, which raised tissue oxygen level in the RVLM to approximately 20 mmHg. We also observed that intravenous administration of CoQ10, which exerts its primary effects on cardiac mitochondria, was less effective in promoting resumption of spontaneous circulation. This observation again reinforces the vital importance of bioenergetics at the RVLM in cardiac resuscitation.

We recognize that since our animal model mimics clinically the progression towards brain stem death in patients who succumbed to organophosphate poisoning [10], [16], ours may be taken as a specific model that is irrelevant to cardiac arrest in general clinical contexts. This concern, however, is not at issue because the primary purpose of cardiac resuscitation is to revive an arrested heart regardless of its etiology. On the other hand, as elaborated under Introduction, our animal model allowed us to delineate the inter-relationship between brain stem death and resumption of spontaneous circulation after cardiac arrest and the underlying cellular mechanisms.

The present study provided novel evidence to support the provocative thesis that a crucial determinant for successful revival of an arrested heart is that spontaneous circulation must resume before brain stem death commences. We demonstrated that this vitally important time-window is dependent on maintained neuronal functionality in the RVLM. We additionally showed that the repertoire of interposing cellular events in the RVLM, which precipitates brain stem death and underpins the failure of resumption of spontaneous circulation after cardiac arrest, entails progression towards sustained anoxia, cessation of local blood flow, dysfunction of mitochondrial electron transport chain and reduction in ATP production, leading to necrotic cell death. Oxidative stress in the RVLM has been shown to underlie cardiovascular depression [29]. The ameliorating effects of CoQ10 and oxygenation identified in this study further suggest the importance of improving the efficacy or capacity of mitochondrial electron transport chain functions in the RVLM in enhancing the resumption of spontaneous circulation after cardiac arrest. These novel findings provide a new vista for future development of therapeutic and management strategies towards resuscitation of cardiac arrest, targeting at improvement of central cardiovascular regulatory efficacy by maintaining the functional integrity of RVLM.
